# To Identify Adenomatous Polyposis Coli Gene Mutation as a Predictive Marker of Endometrial Cancer Immunotherapy

**DOI:** 10.3389/fcell.2022.935650

**Published:** 2022-07-22

**Authors:** Yunfeng Song, Jian Huang, Kai Wang, Yiran Li

**Affiliations:** ^1^ Department of Gynecology, Shanghai First Maternity and Infant Hospital, Tongji University School of Medicine, Shanghai, China; ^2^ Clinical and Translational Research Center, Shanghai First Maternity and Infant Hospital, Tongji University School of Medicine, Shanghai, China; ^3^ Harvard Medical School, Boston, MA, United States

**Keywords:** endometrial cancer (EC), adenomatous polyposis coli (APC), programmed death-ligand 1 (PD-L1), tumor mutational burden (TMB), lymphocytic infiltration, immunotherapy

## Abstract

The adenomatous polyposis coli (APC) gene is the chromatin-remodeling-related gene and a typical tumor suppressor. Patients with a high expression of programmed death-ligand 1 (PD-L1) or a high level of tumor mutational burden (TMB) may benefit from immunotherapy in endometrial cancer (EC). This study aimed to demonstrate the role of APC in the diagnosis and immunotherapy treatment of EC. We performed an integrative analysis of a commercial panel including 520 cancer-related genes on 99 tumors from an endometrial cancer cohort in China and DNA-seq data from The Cancer Genome Atlas (TCGA) to identify new gene mutations as endometrial cancer immunotherapy markers. We found that the significant mutant genes that correlated with the PD-L1 expression and TMB were related to the chromatin state and generated a discovery set having 12 mutated genes, including the APC gene, which was identified as a new marker for immunotherapy. Further analysis revealed that tumors with the APC mutation had high TMB, increased expression of PD-L1, and increased lymphocytic infiltration. Next, we verified that APC has an inactive mutation in EC, which may affect the immune response, including PD-L1 expression, microsatellite instability, and lymphocytic infiltrate. Furthermore, patients with the APC mutation had longer overall survival. Our study demonstrates that APC could play an important role in enhancing the response to endometrial cancer treatment, particularly immunotherapy.

## Introduction

Endometrial cancer is one of the most common malignant tumors of the female reproductive system. In recent years, the morbidity and mortality of endometrial cancer have increased worldwide. Early diagnosis, surgery, and chemotherapy reduce endometrial cancer mortality. However, there is a subset of low-grade, early-stage, well-differentiated endometrioid tumors in which unexpected recurrences and poor outcomes do occur. For women diagnosed with a clinically aggressive histologic subtype of the disease, such as serous EC, clinical outcomes worsen considerably with recurrent or advanced diseases (Walker et al., 2009; Siegel et al., 2018). Traditional endometrial cancer-typing (Bokhman) has limited the predictive value for prognosis because it does not consider the genetic variation and heterogeneity of the tumor. The Cancer Genome Atlas (TCGA) published a comprehensive genomic study of serous and endometrioid histotypes and reported four genomic subtypes: polymerase-epsilon (POLE), microsatellite instability (MSI), copy-number low, and copy-number high (Cancer Genome Atlas Research Network, 2013). Intracellular mutation accumulation caused by POLE mutations or MSI, leads to a high tumor mutational burden (TMB), increased expression of new antigens, and abundant tumor-infiltrating lymphocytes, which result in sensitivity to immune checkpoint inhibitors (Arora et al., 2018).

Immunotherapy, the most promising research direction in cancer treatment, regulates the tumor immune microenvironment to kill tumor cells by activating or regulating the body’s immune system. Multiple clinical trials have proven that the type of POLE hypermutation and MSI are potential beneficiaries of programmed cell death protein (PD-1) and programmed death-ligand 1 (PD-L1) immunotherapies in endometrial cancer (Cancer Genome Atlas Research Network, 2013; Le et al., 2015; Mehnert et al., 2016). The expression of PD-L1 was typically detected by immunohistochemistry to predict the effect of immunotherapy in endometrial cancer. However, some clinical studies have indicated that patients with a positive PD-1 expression do not respond to PD-1/PD-L1 immunotherapy (Mehnert et al., 2016; Le et al., 2017). Coincidentally, the latest research by Dou et al. found that the efficiency of antigen expression varies greatly in the MSI-type endometrial cancer, and the down-regulation of the antigen’s presentation ability would alter the effect of immunotherapy (Le et al., 2017; Dou et al., 2020). It is urgent to identify new targets for diagnosis and treatment to improve the effect of immunotherapy in endometrial cancer.

Epigenetics is the study of heritable phenotype changes, most often involving changes that affect gene activity and expression, but do not involve DNA sequence alterations. Due to epigenetic changes, tumor cell immunogenicity and immune recognition mechanisms are destroyed (Mcdermott et al., 2014; Maio et al., 2015; Wu et al., 2015). In addition, epigenetic silencing affects antigen processing and presentation (Sigalotti et al., 2014). Chromatin remodeling is an important part of epigenetics. Impaired chromatin-remodeling leads to the accumulation of epigenetic abnormalities. Several studies have found that, in malignant tumors, the gene related to chromatin remodeling has a high mutation frequency and plays an important role in tumor immune escape (Miao et al., 2018; Pan et al., 2018). The adenomatous polyposis coli (APC) gene, located on chromosome 5q21, is the chromatin remodeling-related gene and a typical tumor suppressor. APC protein is involved in the modification of transcription activation and cell cycle regulation. APC has an oligomerization domain, a 15- or 20-residue repeat domain important for binding to β-catenin, SAMP repeats for axin binding, a basic domain for microtubule binding and C-terminal domains that bind to EB1 and DLG proteins (Sansom et al., 2004). The basal region and the C-terminal region, combined with microtubules, interact with EB1 to promote chromosome aggregation (Browne et al., 1998). APC inactivation leads to the loss of spindle function in mitosis and the instability of the genome and chromosome (Baeg et al., 1995). An aberrant structure or expression of APC has been reported to be associated with various cancers. For example, a high APC expression is an unfavorable prognostic factor for T4 gastric cancer and may be used as a novel biomarker for pathogenesis research, diagnosis, and the treatment of gastric cancer (Du et al., 2019). Loss of APC function leading to Wnt/β-catenin signaling hyper-activation is considered to be one of the driving forces of colorectal cancer tumorigenesis (Fodde et al., 2001). APC mutation occurs in 20–45% of endometrial cancers (Karbova et al., 2002), and the methylation of APC is associated with endometrial cancer occurrence (Zysman et al., 2002). Recent studies have shown that APC mutation can induce endometrial hyperplasia and endometrial cancer by preventing estrogen signal transduction in the endometrial epithelium (Tanwar et al., 2011). Therefore, we speculated that APC may have an important role in the pathogenesis and clinical progression of endometrial cancer.

The objective of this study was to demonstrate that APC is a new target for the diagnosis and treatment of endometrial cancer. We used data from TCGA and tumor samples from an endometrial cancer cohort to characterize endometrial cancer with and without the APC mutation. And our findings support that APC may play an important role in the immunotherapy of endometrial cancer.

## Materials and Methods

### Research Data Source

We randomly selected 100 endometrial cancer tumors from 1,000 in the endometrial cancer cohort biorepository of Shanghai First Maternity and Infant Hospital. The specimens were kept on dry ice to maintain specimen integrity and then cryo-pulverized. The cryo-pulverized specimen and paired blood samples were prepared for DNA isolation for molecular characterization. Given that one sample failed the quality test, a total of 99 endometrial cancer patients were enrolled in this study, with an average age of 58 years. The cohort study in China includes 88 cases of endometrioid adenocarcinoma, 6 cases of uterine carcinosarcoma, 3 cases of serous carcinoma, and 2 cases of other types of endometrial cancer. Among them, 74.2% of patients were in grade I, followed by19.1% in grade II, and 6.7% in grade III. Most of the samples submitted for inspection were in the early-stage (71.6% for stage I and 10.1% for stage II), and 18.1% in the late-stage. The data from TCGA, including high throughput RNA sequencing (RNA-Seq) and clinical follow-up information for uterine corpus endometrioid carcinoma, were downloaded from TCGA on 31 May 2020. The samples from TCGA with more than 30 days of follow-up were screened for clinical follow-up data to further match the RNA-seq expression profile.

### DNA Extraction and Fragmentation

DNA isolation and subsequent sequencing procedures were performed in the laboratory of Burning Rock Biotech (Guangzhou, China) accredited and certified by the College of American Pathologists and Clinical Laboratory Improvement Amendments. Genomic DNA (gDNA) was extracted from tumor tissues and white blood cells by using the QIAamp DNA Blood Mini Kit (Qiagen, Hilden, Germany). Qubit fluorometer with the dsDNA high-sensitivity assay kit (Life Technologies, Carlsbad, CA, United States) was used to measure DNA quality following the manufacturer’s instructions. DNA fragmentation was performed using a Covaris M220 Focused-ultrasonicator (Woburn, MA, United States), followed by end repair, phosphorylation, and adapter ligation. Fragments between 200 and 400 bp were selected using AMPure beads (Agencourt AMPure XP Kit, Beckman Coulter, Brea, CA, United States). Subsequently, the hybridization with capture probe baits, hybrid selection with magnetic beads, and PCR amplification were performed. A high-sensitivity DNA assay was then performed to assess the quality and size of the DNA fragments (Agilent 2,100 bioanalyzer instrument, Agilent, Santa Clara, CA, United States).

### Next-Generation Sequencing Library Preparation and Capture-Based Targeted DNA Sequencing

The NGS library was constructed for the DNA isolated from tumor tissues and white blood cells according to an optimized protocol. A minimum of 50 ng of DNA was required for the NGS library construction. Target capture was performed using commercially available panels of 520 cancer-related genes (OncoScreen Plus, Burning Rock, Guangzhou, China), spanning 1.64 Mb of the human genome (Burning Rock Biotech, Guangzhou, China). These panels comprehensively and accurately detect single nucleotide variants, insertions–deletions, copy number variations (CNV), and the structural variations of genes that are clinically relevant to cancer. The average sequencing depths were 1,000X for tissue DNA. Indexed samples were sequenced on the Nextseq500 sequencer (Illumina, Inc. San Diego, CA, United States) with paired-end reads. The sequencing data were analyzed using proprietary computational algorithms optimized for somatic variant calling as described previously (Mao et al., 2017; Li et al., 2018).

All data can be viewed in NODE (http://www.biosino.org/node) by pasting the accession (OEP001202) into the text search box or through the URL: http://www.biosino.org/node/project/detail/OEP001202.

### Sequencing Data Analysis

The sequencing data in the FASTQ format were mapped to the human genome (hg19) using Burrows–Wheeler Aligner v.0.7.10 (Li and Durbin, 2009). Local alignment optimization and variant calling were performed using GATK v.3.2 (Mckenna et al., 2010) and VarScan v.2.4.3 (Koboldt et al., 2012), respectively. The variants were filtered using the VarScan fpfilter pipeline; loci with a sequencing depth of less than 100 were eliminated. Matched white blood cells were used to filter out germline mutations. Variant calling in plasma and tissue samples required at least eight supporting reads for single nucleotide variants and two and five supporting reads for insertions and deletions, respectively. The variants with a population frequency over 0.1% in the ExAC, 1,000 Genomes, dbSNP or ESP6500SI-V2 databases were grouped as single nucleotide polymorphisms and excluded from further analyses. The remaining variants were annotated with ANNOVAR (2016-02-01 release) (Wang et al., 2010) and SnpEff v.3.6 (Cingolani et al., 2012). A DNA translocation analysis was performed using Factera v.1.4.3 (Newman et al., 2014). Gene-level CNV was assessed using an in-house-developed algorithm based on a statistic after normalizing the read depth at each region by the total read number and region size and correcting the GC-bias using a LOESS algorithm. CNV was called if the coverage data of the gene region was quantitatively, statistically, and significantly different from its reference control. The limit of detection for CNVs was 1.5 for copy number deletions and 2.64 for copy number amplifications. TMB was calculated as the ratio of mutation count to the size of the coding region of the panel (1.26 Mb), excluding CNV, fusions, large genomic rearrangements, and mutations occurring on the kinase domain of EGFR and ALK. The MSI phenotype detection method used a read-count–distribution-based approach according to a previously published protocol and algorithm (Zhu et al., 2018).

### Immunohistochemistry

Paraffin-embedded tissue sections (4 μm) of the endometrial cancer samples were processed for immunohistochemistry. First, the specimens were deparaffinized and dehydrated, and then the sections were stained with an anti-APC antibody from Abcam 1:100 dilution); anti-PD-L1 antibody from Ventana (prediluted); anti-CD3+antibody and anti-CD8^+^ antibody from Springs (1:200 dilution); and anti-MLH1antibody, anti-MSH2, anti-MSH6, and anti-PMS2 from Maixin (1:100 dilution). After washing, the sections were incubated with biotin-conjugated secondary antibodies and subsequently with streptavidin–HRP. The sections were finally visualized by incubation with a 3,3′diaminobenzidine substrate. Images were obtained with the Mantra System (PerkinElmer, Waltham, Massachusetts, United States) with identical exposure times. The integrated optical density was used to quantify the protein levels of APC, PD-L1, CD3^+^, CD8^+^, MLH1, MSH2, MSH6, and PMS2 in the tumor tissue, and this integrated optical density was calculated by staining intensity by dividing the staining area (brown stained area).

### Statistics

Categorical data were described by frequency and percentage. Quantitative variables were expressed as means ± SEM. Fisher’s exact or Chi-square test was performed to compare categorical variables. The Student t-test was used to compare the continuous variables between two groups. Pearson’s correlation coefficient was used to assess the correlations. All the data were analyzed *via* the R statistics package (R version 3.5.3; R: The R-Project for Statistical Computing, Vienna, Austria). All statistical tests were two-sided, and *p*-values <0.05 were considered significant. The Spearman rank correlation test was used to analyze the correlation between two parameters. R package survival KM (Kaplan–Meier) was used to estimate the survival rate, and Cox proportional hazards regression was used to calculate the hazard ratio.

## Results

### Comprehensive Analysis of Gene Mutation in Endometrial Cancer

**FIGURE 1:**
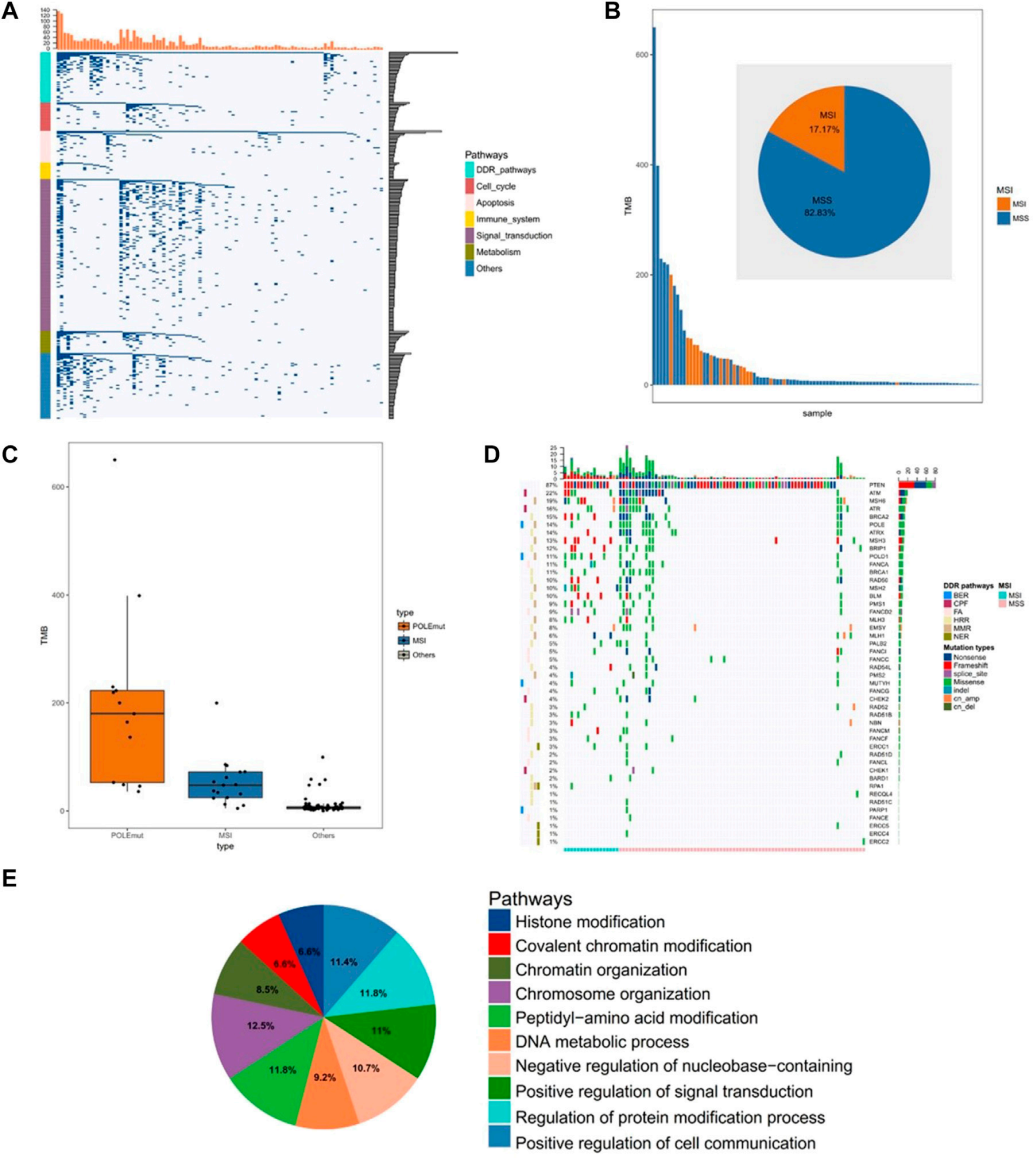
Comprehensive analysis of the gene mutations in endometrial cancer. **(A)** Unsupervised clustering of gene mutations in endometrial cancer. Samples are presented in columns, and the 520 cancer-related genes are presented in rows. **(B)** The analysis of microsatellite instability in 99 samples **(C)** The analysis of tumor mutational burden level in the POLE (DNA polymerase epsilon, catalytic subunit) mutant group, MSI (microsatellite instability) group, and other groups **(D)** The analysis of mutated genes related to the DDR pathway **(E)** The pathway analysis of significantly mutated genes (mutation >10%).

The clinical and pathological characteristics of the 99 tumors from the cohort biorepository are summarized in Supplementary Table S1. We used tissues and peripheral blood to detect gene mutations. The sequencing results showed that the high-frequency mutant genes of somatic cells were PTEN, PIK3CA, and ARID1A, with 82, 62, and 53% mutation rates, respectively. DNA damage repair (DDR) gene mutations were detected in 94% of the samples (Figure 1A). We also found that 17% of tumors had high levels of MSI, and the tumors with MSI had higher TMB (mean TMB: 53.25 mut/mb in MSI, 37.37 mut/mb in the Microsatellite table). (Figure 1B). In addition, we found that TMB was significantly higher in tumors with the POLE mutant and those with MSI than those without them (both *p* < 0.01; Figure 1C). In our study, the total mutation rate of mismatch repair genes, including MSH2, MSH6, MLH1, and PMS2, was 26% (Figure 1D). By analyzing the genes with more than 10% mutations, we found that most of the mutated genes related to chromatin status, including covalent modification of chromatin, chromosome organization, and chromatin organization (Figure 1E).

### Immune Microenvironment Correlating With Chromatin Status-Related Genes

**FIGURE 2:**
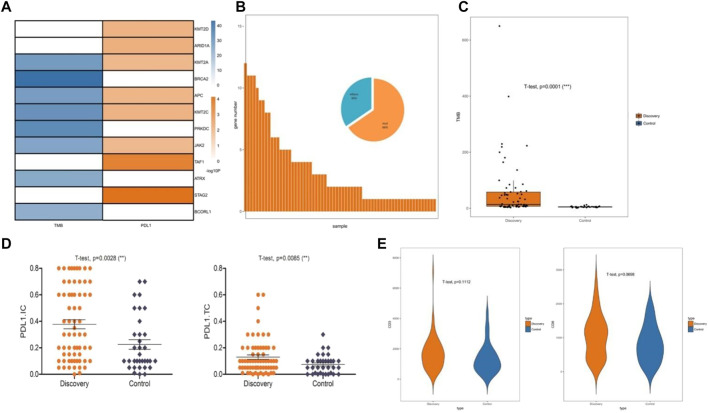
Immune microenvironment correlated with chromatin status-related genes. **(A)** Specific mutated genes screened by significant correlation with the PD-L1 (programmed death-ligand 1) expression or TMB (tumor mutational burden), Pearson’s correlation coefficient was used to assess correlations. **(B)** Mutations of 12 specific mutated genes were identified as a discovery set. **(C)** Relationship between the discovery sets and TMB. **(D)** Relationship between the discovery sets and PD-L1 expression. **(E)** Relationship between the discovery sets and CD3^+^ and CD8^+^ T cell infiltration.

To determine the effect of chromatin-related gene mutations on endometrial cancer, we explored the correlation between gene mutations and a tumor immune microenvironment. From the mutated chromatin-related genes, we identified 12 mutant genes that were significantly positively correlated with the PD-L1 expression (R > 0.25; *p* < 0.05) or TMB (R > 0.8; *p* < 0.05). Mutations of these 12 genes (STAG2, TAF1, ARID1A, KMT2C, KMT2D, APC, KMT2A, JAK2, BRCA2, PRKDC, ATRX, and BCORL1) were detected in 66% of the endometrial cancer samples (Figures 2A,B). The 66 samples that contained these mutated genes comprised the discovery set, and the remaining 33 samples were used as the control set. Next, we compared the discovery set with the control set in terms of TMB, PD-L1 expression, and lymphocytic infiltration. The mean TMB of the discovery set (58.57 mut/mb, log2TMB = 4.45; SD = 1.96) was significantly higher than that of the control set (4.92 mut/mb, log2TMB = 2.16; SD = 0.64; *p* < 0.001; Figure 2C). We then quantified the expression of PD-L1 in tumor cells and immune cells. The PD-L1 expression was higher in the discovery set than in the control set in both the immune cells and tumor cells. The PD-L1 expression increased significantly (both *p* = 0.001), especially in the immune cells (PDL1. TC: 0.074 in control and 0.130 in discovery; PDL1. IC: 0.225 in control and 0.378 in discovery; Figure 2D). When comparing the discovery set and the control set, we found no significant difference in lymphocytic infiltration for CD3^+^ T cells (*p* = 0.11) or CD8^+^ T cells (*p* = 0.07; Figure 2E).

### Chromatin Remodeling-Related Gene Adenomatous Polyposis Coli Affects the Immune Microenvironment

**FIGURE 3:**
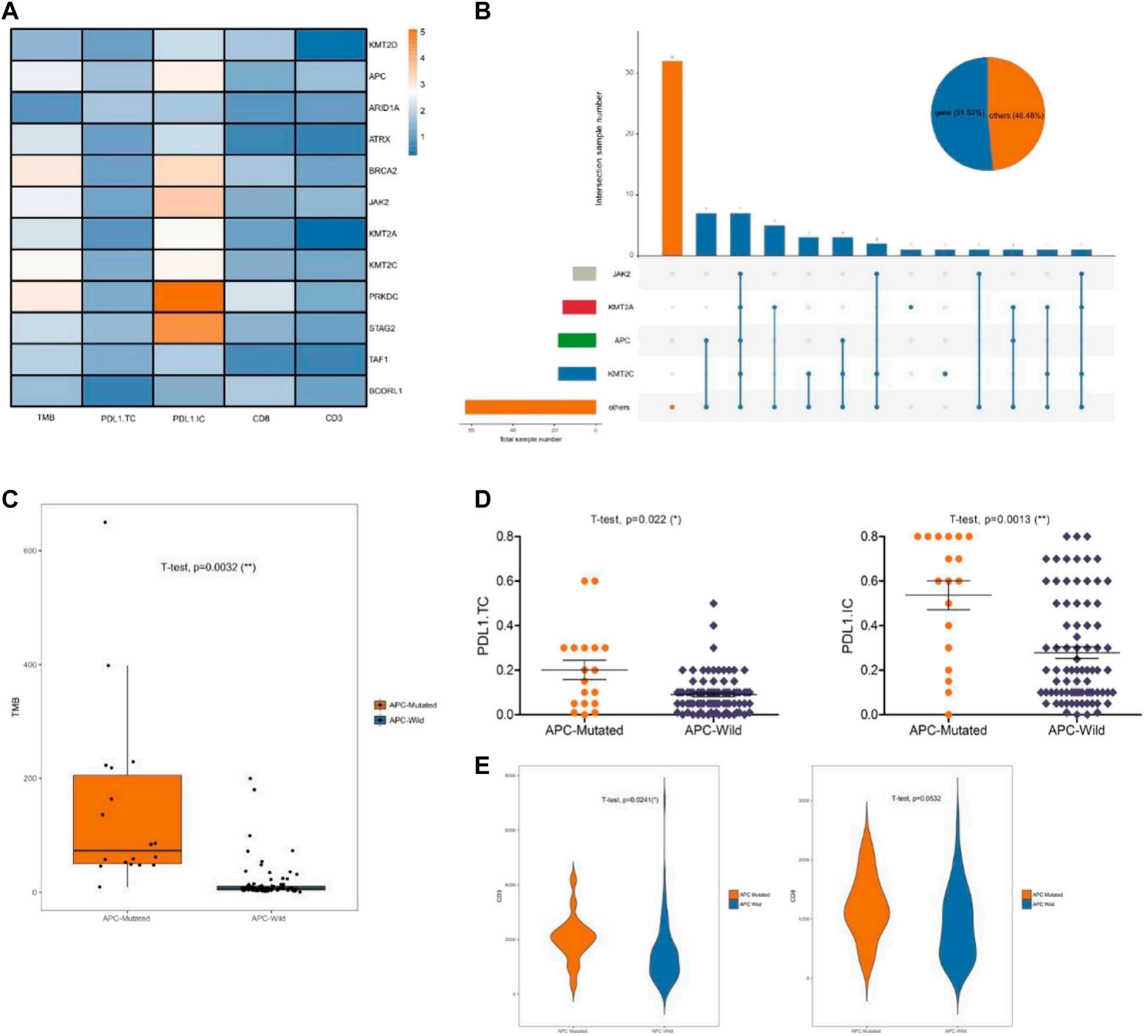
Chromatin remodeling-related gene APC affects the immune microenvironment. **(A)** Significant change of TMB, PD-L1 expression, and lymphocytic infiltration inducing by the mutations of the discovery sets. **(B)** The mutations of KMT2C, APC, KMT2A, and JAK2. **(C)**. Relationship between APC and TMB. **(D)** Relationship between APC and PD-L1 expression. **(E)** Relationship between APC and CD3^+^ and CD8^+^ T cell infiltration.

**FIGURE 4:**
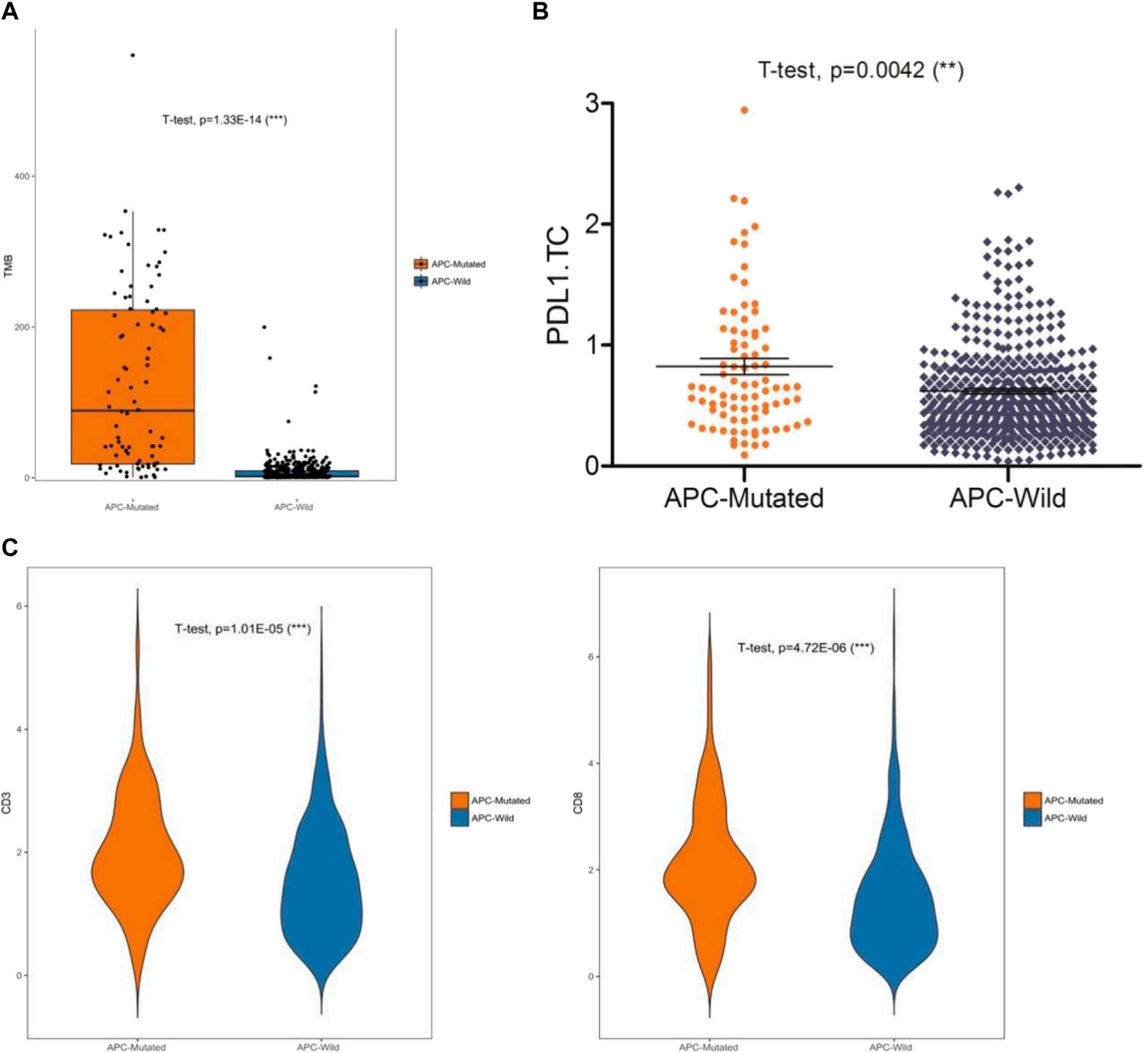
Analysis of data in the TCGA database. **(A)** Relationship between APC and TMB **(B)** Relationship between APC and PD-L1 expression. **(C)** Relationship between APC and CD3^+^ and CD8^+^ T cell infiltrations.

To evaluate the effects of the 12 specific gene mutations, we explored the immune microenvironment, including TMB, PD-L1 expression, and lymphocytic infiltration in endometrial cancer (Figure 3A). Four genes (KMT2C, APC, KMT2A, and JAK2) were selected for significance and relevance. The mutation rates were 18% for KMT2C, 18% for APC, 17% for KMT2A, and 11% for JAK2 (Figure 3B). Unlike the other three genes, the mutations of the chromatin remodeling-related gene APC were associated with significant increases in TMB, PD-L1 expression, and CD3^+^ T cell infiltration (all *p* < 0.02; Figures 3C–E). To further verify the impact of APC mutations on the immune microenvironment, we analyzed the gene mutations of endometrial cancer from TCGA, which yielded similar results (Figures 4A–C).

### Subtypes of Chromatin Remodeling-Related Gene Adenomatous Polyposis Coli

**FIGURE 5:**
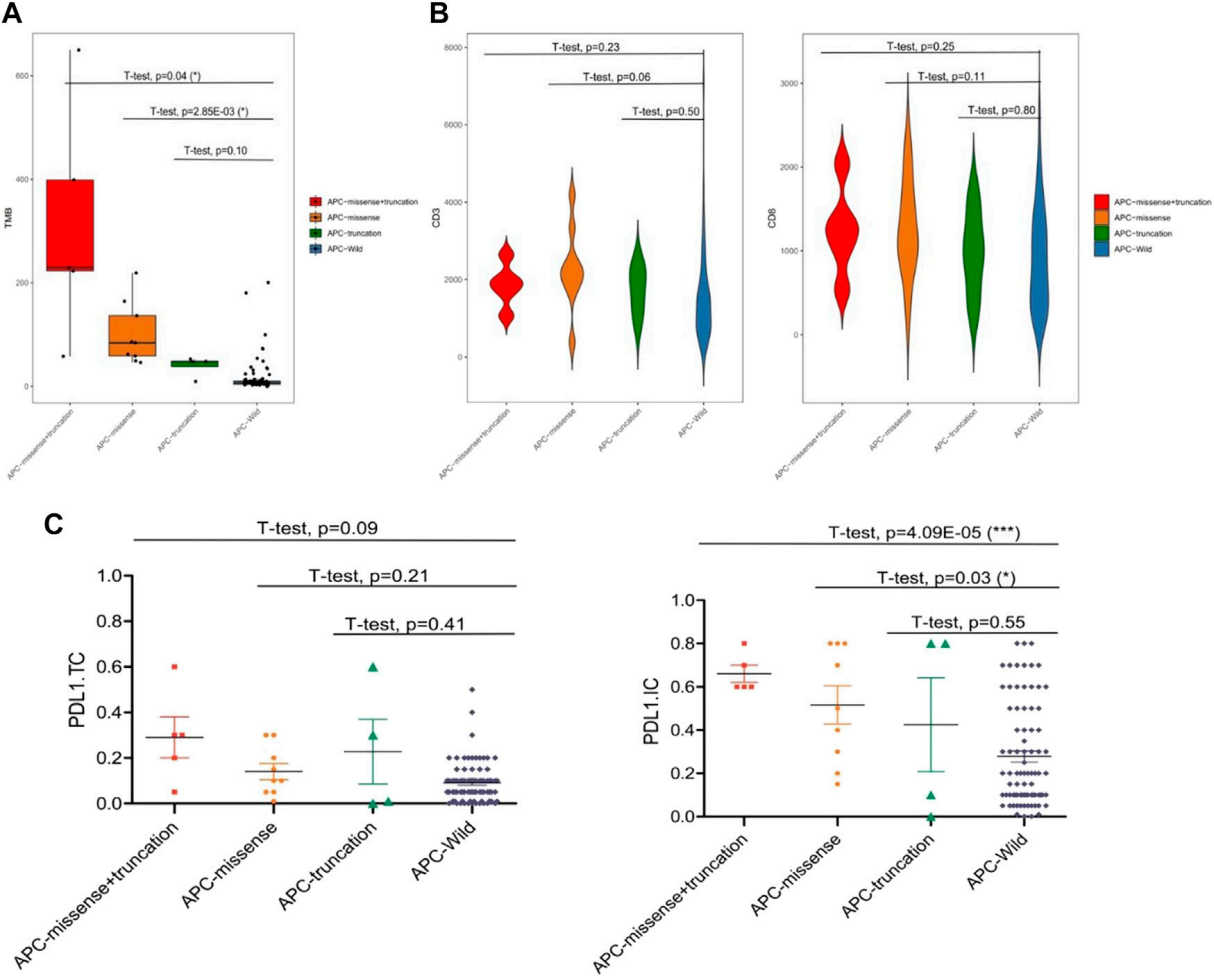
Characteristic of the APC mutation in the SFMIH cohort. **(A)** Level of TMB in the subtypes of APC. **(B)** CD3^+^ and CD8^+^ T cell infiltrations in the subtypes of APC. **(C)** PD-L1 expression in the subtypes of APC.

**FIGURE 6:**
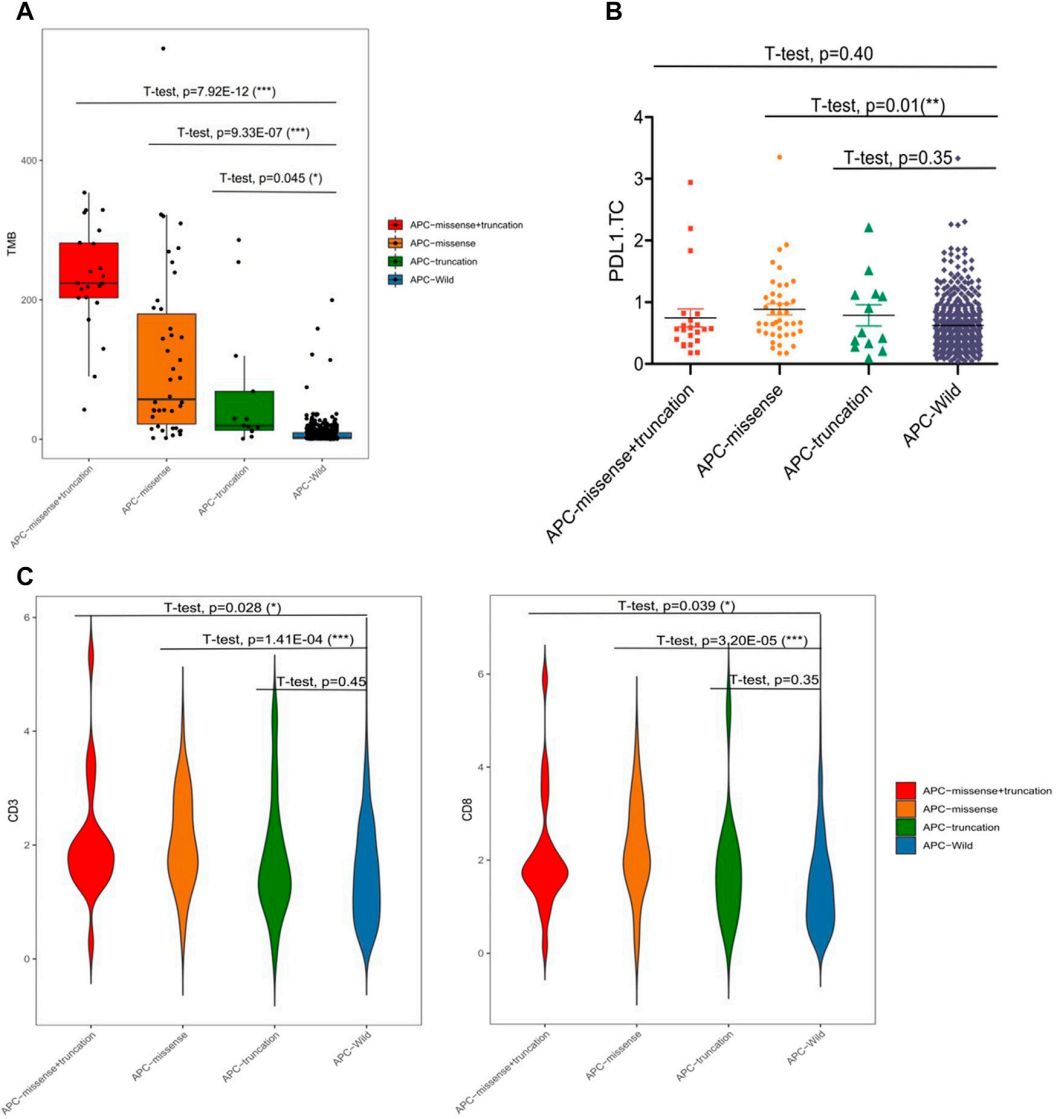
Characteristic of APC mutation in the TCGA cohort. **(A)** Level of TMB in subtypes of APC. **(B)** PD-L1 expression in subtypes of APC. **(C)** CD3^+^ and CD8^+^ T cell infiltrations in the subtypes of APC.

Among the 99 samples in the SMFIH cohort, there were 18 cases of APC mutations, including 9 cases of missense mutations, 4 cases of truncating mutations, and 5 cases of a mixture of missense mutations and truncating mutations. The 86 cases of APC mutations were detected in 526 samples of the TCGA cohort, which contained 42 cases of missense mutations, 13 cases of truncating mutations, 22 cases of a mixture of the two mutations, and 9 cases of other mutation types. We have separately verified whether the impact of the APC gene on the tumor immune microenvironment depends on the mutation pattern in the two cohorts. In the SFMIH cohort, APC missense mutations were accompanied by high levels of TMB and an increased expression of PD-L1 in tumor-infiltrating immune cells (ICs) (Figures 5A–C). However, no matter which mutation type of APC, the infiltration of T lymphocytes did not change significantly (Figure 5B). Whereas, the APC missense mutation group had a high level of TMB, increased expression of PD-L1, and rising infiltration of T lymphocytes in the TCGA cohort (Figures 6A–C). In summary, due to the increased immune response ability of the tumor microenvironment, immunotherapy may be more effective for patients with APC missense mutations. Since having fewer samples with other types of APC mutations, including truncating mutation, the tumor immune microenvironment is not significantly changed.

### Chromatin Remodeling-Related Gene Adenomatous Polyposis Coli Expression in Endometrial Cancer

**FIGURE 7:**
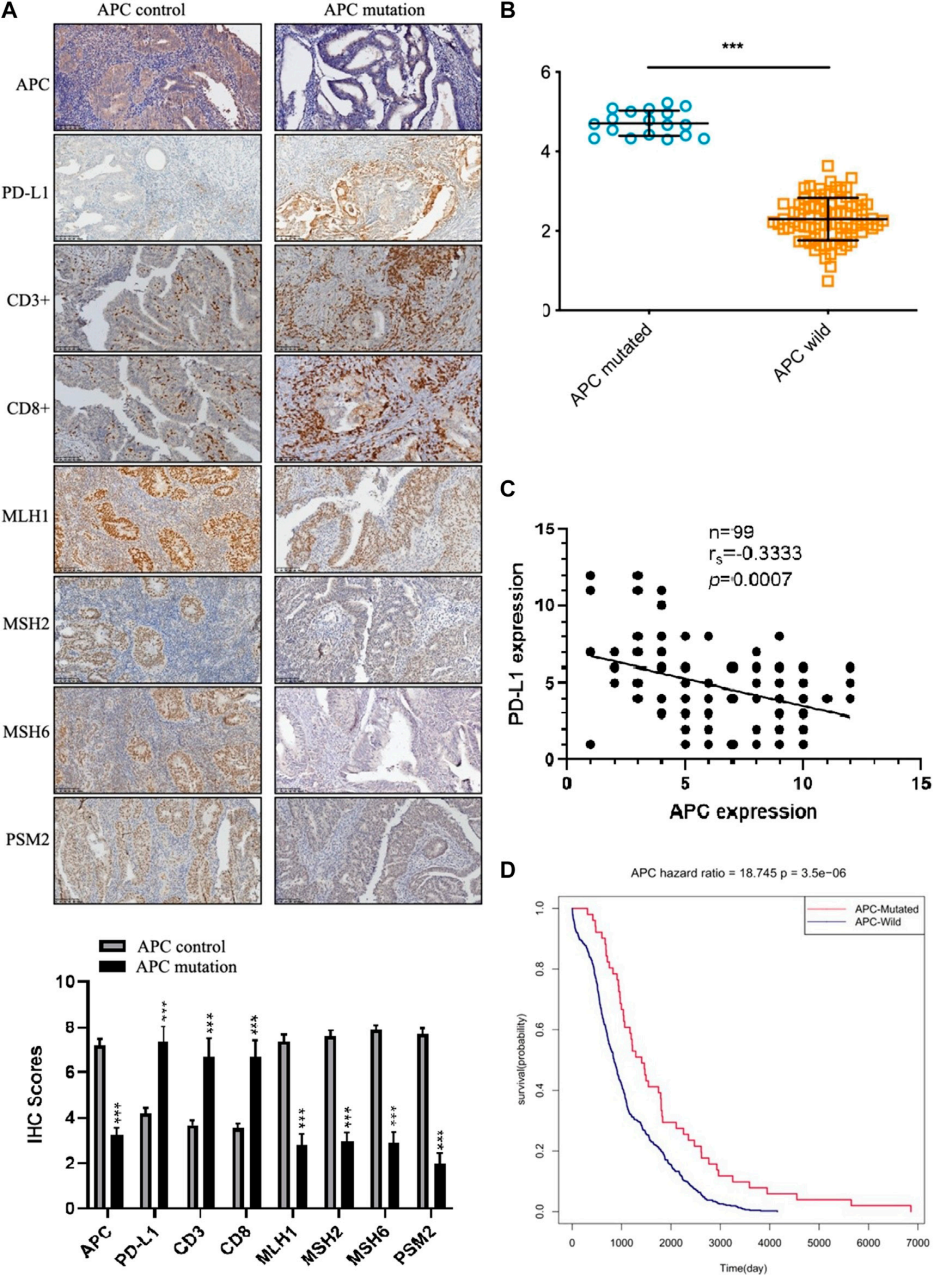
The expression of APC in endometrial cancer. **(A)** Immunohistochemistry staining of the indicated proteins in APC control samples and APC mutation samples. **(B)** The APC gene is weakly expressed in human endometrial cancer (*p* = 6.17624e-27). **(C)** The Spearman rank correlation analysis of the correlation between the PD-L1 expression and APC expression (*n* = 99, *p* = 0.0007). **(D)** Prognostic value of the APC expression in endometrial cancer patients from TCGA (Kaplan–Meier was used to estimate the survival rate).

To further investigate the expression of the APC endometrial cancer-specific immunotherapy markers, we assessed the protein levels of APC, MLH1, MSH2, MSH6, PMS2, PD-L1, CD3^+^, and CD8^+^ by immunohistochemistry using tissues from 99 endometrial cancer tumors. We quantified staining with the integrated optical density value that combined the staining intensity and the percentage of positive cells. In endometrial cancer, most APC mutations are inactive mutations that lead to reduced protein levels (Tanwar et al., 2011). In our study, the low expression of APC was accompanied by high levels of the PD-l expression and increased infiltration of CD3^+^ and CD8^+^ T cells (Figure 7A). To evaluate MSI, we quantified the expression levels of MLH1, MSH2, MSH6, and PMS2 by immunohistochemistry. The results indicated that samples with APC inactivated mutations were of the MSI type, which was consistent with gene sequencing (Figures 7A, B). The Spearman rank correlation analysis showed that the PD-L1 expression was negatively correlated with APC expression (*n* = 99, *p* = 0.0007; Figure 7C). Moreover, the results of the survival analysis among the 526 TCGA samples suggested that the APC mutation was associated with a longer survival (*p* = 3.5e-06; Figure 7D).

## Discussion

We analyzed the gene expression profiles in endometrial cancer samples from our cohort by using a commercial panel of 520 genes that are closely associated with cancer pathogenesis. We comprehensively analyzed the mutation profiles of endometrial cancer and confirmed the somatic and germline mutations of the previous study described at the genomic level (Cancer Genome Atlas Research Network., 2013). In recent years, PD-1/PD-L1 treatment that blocks immune checkpoints has developed rapidly and attracted attention broadly. TMB, MSI, and PD-L1 expressions all have been reported as markers for PD-1/PD-L1 immunotherapy (Hause et al., 2016; Mehnert et al., 2016; Arora et al., 2018). However, as a result of paradoxical clinical trials (Le et al., 2017), the issue has been the identification of markers that accurately predict the efficacy of immunotherapy in endometrial cancer. Our results revealed that the samples characterized by MSI or POLE mutations were accompanied by an increase in TMB. Beyond that, the clustering analysis of gene mutation profiles showed that 94% of the samples had mutations of the DDR gene, and we demonstrated that genes with more than 10% mutations are related to the chromatin status. These findings suggested that mutations of chromatin state-related genes may have an impact on the immune microenvironment of endometrial cancer.

Feature selection is a data preprocessing technique that has been widely used in many bioinformatics applications (Saeys et al., 2007). Here, we modeled the marker discovery as an approach to select the best feature subset for the immunotherapy of endometrial tumors. It is not easy to choose a reliable feature subset due to the multi-dimensionality of the QIAamp DNA FFPE tissue kit data. Therefore, we designed a cross-feature selection schema, based on the correlation of chromatin status-related genes with the PD-L1 expression or TMB, to select a reliable subset. Filters may lead to locally optimum sets but not the best discriminative subset, which may make it impossible to find diagnostic markers with high sensitivity and specificity. We further analyzed the TMB level, PD-L1 expression, and lymphocytic infiltration of the selected subset to verify its reliability, which was identified to contain 12 chromatin state-related genes. The results indicated that the cross method performed extremely well for identifying a discovery set of genes associated with the immune microenvironment in endometrial cancer, except for lymphocytic infiltration.

As cancer treatments, immunotherapy approaches have been highly successful but can be affected by the immune microenvironment. Our results indicate that the gene APC may serve as a new marker for assessing the impact on the immune microenvironment. By analyzing the relationship between individual mutant genes in the discovery set and the immune microenvironment, we found that the APC mutations were significantly associated with the immune microenvironment, including TMB, PD-L1 expression, and lymphocytic infiltration. Our results showed that the APC mutations might suggest elevation of the TMB, PD-L1 expression, and lymphocytic infiltration, which may help identify patients who may benefit from immunotherapy. Moreover, we confirmed our findings regarding the effect of the APC mutations on the immune-microenvironment using TCGA. Further biological validation and clinical trials are needed to evaluate the clinical significance of chromatic status-related gene APC, along with studies to understand the mechanism by which APC regulates the immune microenvironment.

In our study, the significantly decreased expression of APC was further confirmed in human endometrial cancer tissues by immunohistochemistry. The APC mutations may contribute to increased expressions of new antigens and abundant tumor-infiltrating lymphocytes, which may result in the sensitivity of immune checkpoint inhibitors. This may explain why patients with APC mutations have a more favorable prognosis.

## Conclusion

In summary, immune therapy, including checkpoint inhibition and tumor vaccination, plays a crucial role in cancer treatment. The sensitivity of existing target molecules is insufficient, such that a significant proportion of patients fail to respond to immunotherapy. Our results suggest that APC may affect the immune microenvironment of endometrial cancer; thus, patients with APC mutations may be more sensitive to immunotherapy. Consequently, this information could improve the selection of endometrial cancer patients who will have a better response to immune checkpoint therapy, and thus, a better prognosis. Although our results are observational, they provide the basis for multiple hypotheses of clinical relevance that can and should be further explored by the scientific community.

## Data Availability

The datasets presented in this study can be found in online repositories. The names of the repository/repositories and accession number(s) can be found in the article/Supplementary Material.

## Abbreviations

APC, adenomatous polyposis coli; DDR, DNA damage repair; EC, Endometrial cancer; MSI, Microsatellite instability; PD-1, Programmed cell death protein; PD-L1, Programmed death-ligand one; POLE, Polymerase-epsilon; TMB, Tumor mutational burden; TCGA, The Cancer Genome Atlas.
