# Supplementary material for: To Identify Adenomatous Polyposis Coli Gene Mutation as a Predictive Marker of Endometrial Cancer Immunotherapy
Source: Front Cell Dev Biol. 2022 Jul 22;10:935650. doi: 10.3389/fcell.2022.935650 (PMC9354690; doi:10.3389/fcell.2022.935650)
Supplement: Supplementary file 1 [file DataSheet1.PDF]

sample	age	Age	Histology	grade	Clinical stage	Clinical stage_1
RS1845085FFP	65	≥55	Endometrioid	3	III	2
RS1842071FFP	57	≥55	Endometrioid	1	II	1
RS1842068FFP	63	≥55	Endometrioid	1	IV	2
RS1842067FFP	52	<55	Endometrioid	1	I	1
RS1842066FFP	51	<55	Endometrioid	1	III	2
RS1842063FFP	51	<55	Endometrioid	2	I	1
RS1842061FFP	68	≥55	Endometrioid	1	I	1
RS1842060FFP	61	≥55	Endometrioid	3	I	1
RS1842058FFP	65	≥55	Endometrioid	1	I	1
RS1842056FFP	62	≥55	Endometrioid	1	I	1
RS1839223TIS	39	<55	Endometrioid	1	II	1
RS1839221TIS	51	<55	Endometrioid	2	I	1
RS1839219TIS	48	<55	Endometrioid	1	I	1
RS1839217TIS	73	≥55	Endometrioid	1	I	1
RS1839215TIS	51	<55	Endometrioid	1	I	1
RS1839213TIS	52	<55	Endometrioid	1	I	1
RS1839210TIS	79	≥55	Endometrioid	1	I	1
RS1839207TIS	54	<55	Endometrioid	1	I	1
RS1839196TIS	58	≥55	Endometrioid	1	III	2
RS1839192TIS	62	≥55	Endometrioid	1	I	1
RS1839190TIS	68	≥55	Endometrioid	2	I	1
RS1839182TIS	53	<55	Endometrioid	2	III	2
RS1839173TIS	65	≥55	Endometrioid	2	I	1
RS1839170TIS	44	<55	Endometrioid	3	III	2
RS1836675TIS	62	≥55	Endometrioid	2	III	2
RS1836673TIS	62	≥55	Endometrioid	2	I	1
RS1836670TIS	59	≥55	Endometrioid	1	I	1
RS1836669TIS	45	<55	Carcinosarcoma	3	III	2
RS1836668TIS	65	≥55	Endometrioid	1	I	1
RS1836667TIS	73	≥55	Serous	3	III	2
RS1836666TIS	63	≥55	Endometrioid	1	I	1
RS1836664TIS	61	≥55	Endometrioid	1	I	1
RS1836663TIS	65	≥55	Endometrioid	1	I	1
RS1836662TIS	64	≥55	Endometrioid	1	I	1
RS1836661TIS	52	<55	Endometrioid	1	I	1
RS1836658TIS	57	≥55	Endometrioid	1	I	1
RS1836655TIS	36	<55	Carcinosarcoma	3	III	2
RS1836654TIS	44	<55	Endometrioid	1	III	2
RS1836653TIS	61	≥55	Endometrioid	1	I	1
RS1836651TIS	69	≥55	Carcinosarcoma	3	I	1
RS1836650TIS	59	≥55	Endometrioid	1	II	1
RS1836647TIS	52	<55	Endometrioid	1	I	1
RS1836644TIS	35	<55	Endometrioid	1	I	1
RS1836643TIS	44	<55	Mixed carcinomas	3	I	1
RS1836642TIS	48	<55	Endometrioid	1	I	1
RS1836641TIS	55	≥55	Serous	3	III	2
RS1836636TIS	51	<55	Endometrioid	3	I	1
RS1836635TIS	46	<55	Endometrioid	1	I	1
RS1836634TIS	60	≥55	Endometrioid	1	I	1
RS1836633TIS	48	<55	Endometrioid	3	II	1
RS1836631TIS	62	≥55	Endometrioid	1	I	1
RS1836627TIS	49	<55	Endometrioid	1	I	1

RS1836622TIS	62	≥55	Endometrioid	1	I	1
RS1836620TIS	67	≥55	Endometrioid	1	I	1
RS1836612TIS	66	≥55	Endometrioid	1	I	1
RS1836609TIS	61	≥55	Carcinosarcoma	3	III	2
RS1836607TIS	54	<55	Endometrioid	1	II	1
RS1836606TIS	52	<55	Endometrioid	2	II	1
RS1836605TIS	48	<55	Endometrioid	1	II	1
RS1836603TIS	72	≥55	Carcinosarcoma	3	I	1
RS1836601TIS	52	<55	Endometrioid	1	II	1
RS1832233TIS	64	≥55	Endometrioid	1	I	1
RS1832232TIS	50	<55	Endometrioid	3	I	1
RS1832231TIS	58	≥55	Endometrioid	1	I	1
RS1832226TIS	59	≥55	Endometrioid	1	III	2
RS1832225TIS	55	≥55	Endometrioid	1	I	1
RS1832223TIS	63	≥55	Clear cell	3	III	2
RS1832222TIS	57	≥55	Endometrioid	1	III	2
RS1832221TIS	64	≥55	Endometrioid	2	I	1
RS1832220TIS	63	≥55	Serous	3	I	1
RS1832217TIS	55	≥55	Endometrioid	1	I	1
RS1832182TIS	70	≥55	Endometrioid	1	I	1
RS1832177TIS	48	<55	Endometrioid	1	I	1
RS1832175TIS	62	≥55	Carcinosarcoma	3	IV	2
RS1832172TIS	59	≥55	Endometrioid	1	I	1
RS1832171TIS	59	≥55	Endometrioid	1	I	1
RS1832169TIS	63	≥55	Endometrioid	2	I	1
RS1827206TIS	63	≥55	Endometrioid	1	I	1
RS1827201TIS	56	≥55	Endometrioid	1	I	1
RS1827200TIS	54	<55	Endometrioid	1	I	1
RS1827199TIS	56	≥55	Endometrioid	1	I	1
RS1827197TIS	56		Endometrioid	2	I	1
RS1827194TIS	63	≥55	Endometrioid	1	I	1
RS1827193TIS	51	<55	Endometrioid	2	I	1
RS1827189TIS	49	<55	Endometrioid	1	III	2
RS1827187TIS	73	≥55	Endometrioid	1	I	1
RS1827184TIS	39	<55	Endometrioid	2	I	1
RS1827148TIS	44	<55	Endometrioid	1	I	1
RS1824041TIS	50	<55	Endometrioid	1	I	1
RS1824039TIS	58	≥55	Endometrioid	2	I	1
RS1824029TIS	65	≥55	Endometrioid	1	II	1
RS1824027TIS	51	<55	Endometrioid	1	I	1
RS1824023TIS	74	≥55	Endometrioid	1	II	1
RS1824021TIS	29	<55	Endometrioid	1	I	1
RS1824019TIS	54	<55	Endometrioid	1	I	1
RS1824017TIS	69	≥55	Endometrioid	2	I	1
RS1824015TIS	62	≥55	Endometrioid	1	I	1
RS1824002TIS	59	≥55	Endometrioid	2	I	1
RS1823991TIS	58	≥55	Endometrioid	1	I	1

Myometrial invasion	Nodal status	Lymph-vascular space invasion	Treatment	gene
≥50%	Unknown	YES	Both	mut
≥50%	Negative	NO	No treatment	mut
≥50%	Unknown	YES	Chemotherapy	normal
≥50%	Negative	NO	No treatment	mut
≥50%	Positive	YES	No treatment	normal
<50%	Negative	NO	No treatment	normal
<50%	Negative	NO	No treatment	mut
<50%	Negative	NO	No treatment	mut
<50%	Negative	NO	No treatment	mut
⩾50%	Negative	NO	No treatment	mut
<50%	Negative	NO	Radiotherapy	normal
<50%	Negative	NO	No treatment	normal
<50%	Negative	NO	No treatment	mut
≥50%	Negative	NO	No treatment	mut
<50%	Negative	NO	No treatment	normal
<50%	Negative	NO	No treatment	normal
<50%	Negative	NO	No treatment	mut
<50%	Negative	NO	No treatment	mut
≥50%	Negative	NO	Both	mut
<50%	Negative	NO	No treatment	mut
≥50%	Negative	YES	No treatment	mut
<50%	Positive	YES	Both	mut
<50%	Negative	NO	No treatment	mut
≥50%	Positive	YES	Chemotherapy	normal
≥50%	Unknown	YES	Chemotherapy	mut
<50%	Negative	NO	No treatment	mut
<50%	Negative	NO	No treatment	normal
≥50%	Negative	NO	Both	normal
≥50%	Negative	NO	No treatment	mut
≥50%	Positive	YES	Chemotherapy	normal
≥50%	Negative	NO	No treatment	mut
<50%	Negative	NO	No treatment	mut
≥50%	Negative	NO	No treatment	mut
<50%	Negative	NO	No treatment	normal
<50%	Negative	NO	No treatment	mut
<50%	Negative	YES	No treatment	mut
≥50%	Positive	YES	Chemotherapy	mut
<50%	Positive	YES	Both	mut
<50%	Unknown	NO	No treatment	mut
≥50%	Negative	NO	Chemotherapy	normal
<50%	Negative	NO	No treatment	mut
<50%	Negative	NO	No treatment	mut
<50%	Negative	NO	No treatment	mut
<50%	Negative	NO	No treatment	normal
<50%	Negative	NO	No treatment	normal
≥50%	Positive	NO	No treatment	mut
<50%	Negative	NO	No treatment	mut
<50%	Negative	NO	No treatment	mut
<50%	Negative	NO	No treatment	normal
≥50%	Negative	NO	Radiotherapy	mut
<50%	Negative	NO	No treatment	mut
<50%	Negative	NO	No treatment	mut

<50%	Negative	NO	No treatment	mut
≥50%	Negative	NO	No treatment	normal
<50%	Negative	NO	No treatment	mut
<50%	Negative	NO	Chemotherapy	mut
<50%	Negative	NO	No treatment	normal
<50%	Negative	NO	No treatment	normal
<50%	Negative	NO	No treatment	normal
≥50%	Negative	NO	Chemotherapy	mut
≥50%	Negative	YES	No treatment	mut
⩾50%	Negative	NO	No treatment	mut
<50%	Negative	NO	Radiotherapy	mut
<50%	Negative	NO	No treatment	mut
⩾50%	Positive	NO	Both	normal
<50%	Negative	NO	No treatment	mut
⩾50%	Positive	NO	Chemotherapy	normal
<50%	Positive	NO	Both	mut
⩾50%	Negative	NO	Chemotherapy	mut
⩾50%	Negative	NO	Chemotherapy	mut
<50%	Negative	NO	No treatment	normal
≥50%	Negative	NO	No treatment	normal
<50%	Negative	NO	No treatment	normal
<50%	Unknown	YES	Chemotherapy	normal
<50%	Negative	NO	No treatment	mut
<50%	Negative	NO	No treatment	mut
<50%	Negative	NO	No treatment	mut
<50%	Negative	NO	No treatment	normal
<50%	Negative	NO	No treatment	normal
<50%	Negative	NO	No treatment	mut
<50%	Negative	NO	No treatment	mut
<50%	Negative	NO	No treatment	mut
<50%	Negative	NO	No treatment	mut
<50%	Negative	NO	No treatment	normal
<50%	Positive	YES	Both	mut
<50%	Negative	NO	No treatment	normal
<50%	Negative	NO	No treatment	normal
<50%	Negative	NO	No treatment	normal
<50%	Negative	NO	No treatment	mut
<50%	Negative	NO	No treatment	mut
⩾50%	Negative	NO	No treatment	mut
<50%	Negative	NO	No treatment	mut
≥50%	Negative	NO	No treatment	mut
<50%	Negative	NO	No treatment	normal
<50%	Negative	NO	No treatment	mut
<50%	Negative	YES	No treatment	mut
⩾50%	Negative	NO	Chemotherapy	normal
<50%	Negative	YES	Chemotherapy	mut
⩾50%	Negative	NO	No treatment	mut
